# GC-MS Fingerprinting Combined with Chemometric Methods Reveals Key Bioactive Components in *Acori Tatarinowii Rhizoma*

**DOI:** 10.3390/ijms18071342

**Published:** 2017-07-03

**Authors:** Wenbin Liu, Bingyang Zhang, Zhongquan Xin, Dabing Ren, Lunzhao Yi

**Affiliations:** 1 Yunnan Food Safety Research Institute, Kunming University of Science and Technology, Kunming 650500, China; lwb93119@163.com (W.L.); xzq220120@163.com (Z.X.); rendabing425@163.com (D.R.); 2 School of Science, Kunming University of Science and Technology, Kunming 650500, China; bingyangzh@126.com

**Keywords:** *acori tatarinowii rhizoma*, radical scavenging activity, gas chromatography–mass spectrometry, partial least squares regression

## Abstract

This present study aims to identify the key bioactive components in *acorus tatarinowii rhizoma* (ATR), a traditional Chinese medicine (TCM) with various bioactivities. Partial least squares regression (PLSR) was employed to describe the relationship between the radical scavenging activity and the volatile components. The PLSR model was improved by outlier elimination and variable selection and was evaluated by 10-fold cross-validation and external validation in this study. Based on the PLSR model, eleven chemical components were identified as the key bioactive components by variable importance in projection. The final PLS regression model with these components has good predictive ability. The *Q*^2^ was 0.8284, and the root mean square error for prediction was 2.9641. The results indicated that the eleven components could be a pattern to predict the radical scavenging activity of ATR. In addition, we did not find any specific relationship between the radical scavenging ability and the habitat of the ATRs. This study proposed an efficient strategy to predict bioactive components using the combination of quantitative chromatography fingerprints and PLS regression, and has potential perspective for screening bioactive components in complex analytical systems, such as TCM.

## 1. Introduction

*Acori tatarinowii rhizoma* (ATR) is a traditional Chinese medicine (TCM) used for thousands of years because of its low toxicity and various bioactivities. Many studies reported the significant clinical effect of ATR for the treatment of diseases, such as epilepsy [1,2] nervous disorders [3,4,5,6], depression [4], cancers [7], skin diseases [8], and Alzheimer’s disease [9]. The research on ATR mainly focuses on its antioxidant activity. Some reports suggested that antioxidants can scavenge free radicals and reduce lipid peroxidation, protein peroxidation, and DNA damage of free radicals [10,11]. The studies on the antioxidants in food, plant materials, and TCMs recently attracted increasing attention [12,13]. However, most studies only evaluated the antioxidant activity of plant extracts or certain chemical components; whether these components are the key antioxidants in the extracts remains poorly understood. Many reports have proven the antioxidant activities of volatile oils in ATR [14]. However, the specific components responsible for these bioactivities remain unknown.

Volatile oils have complex chemical compounds [13], consisting of phenylpropanoid, sesquiterpene, oxygenated-sesquiterpene, monoterpene, and oxygenated-monoterpene [15]. Chromatographic fingerprint is used to characterize the complex chemical composition of TCMs [16,17]. This method was introduced by the World Health Organization to control the quality of TCMs and overcome the limitations when using few marker components [18,19]. Techniques, such as gas chromatography–mass spectrometry (GC-MS) [20], high performance liquid chromatography–diode array detector/mass spectrometry (HPLC-DAD/MS) [21,22], and high performance capillary electrophoresis–mass spectrometry (CE-MS) [23], are commonly used to measure the chromatographic fingerprints of TCMs. In this study, GC-MS fingerprinting was used to measure the complexity of volatile components in ATR. 

Evaluating the bioactivities of each component of ATR volatiles is difficult due to its complexity. Chemometrics became popular because it can deal with groups of variables or discover sets of related predictors. Partial least squares regression (PLSR) [24,25], network-induced supervised learning [26], support vector machine [27], and some penalized methods were proposed for data analysis of complex analytical systems [28]. Multivariate calibration techniques are used to extract information from high throughput analytical data. For example, Liu et al. revealed the antioxidant components in Turpiniae Folium (TF) through PLSR using the information of multi-wavelength fingerprints generated by HPLC and the antioxidant capacity of TFs [18]. These techniques can help reveal the chemical features of TCMs with minimum sample preparation, together with reasonable accuracy and precision without expensive and time-consuming preliminary separation steps, which are usually required for complex systems [29,30,31]. 

This study aims to identify the key bioactive components in ATR for radical scavenging activity. The volatile components in the deliberately collected 49 ATR samples were analyzed by GC-MS. The bioactivity of the volatile oils in ATR was evaluated by 2,2-diohenyl-1-picryl-hydrazyl (DPPH) radical scavenging assay. Furthermore, a PLS regression model between the radical scavenging activity and the contents of volatile components was established and evaluated by using 10-fold cross-validation and external validation. 

## 2. Results and Discussion

### 2.1. GC-MS Fingerprinting and DPPH Radical Scavenging Assay

**Figure 1:**
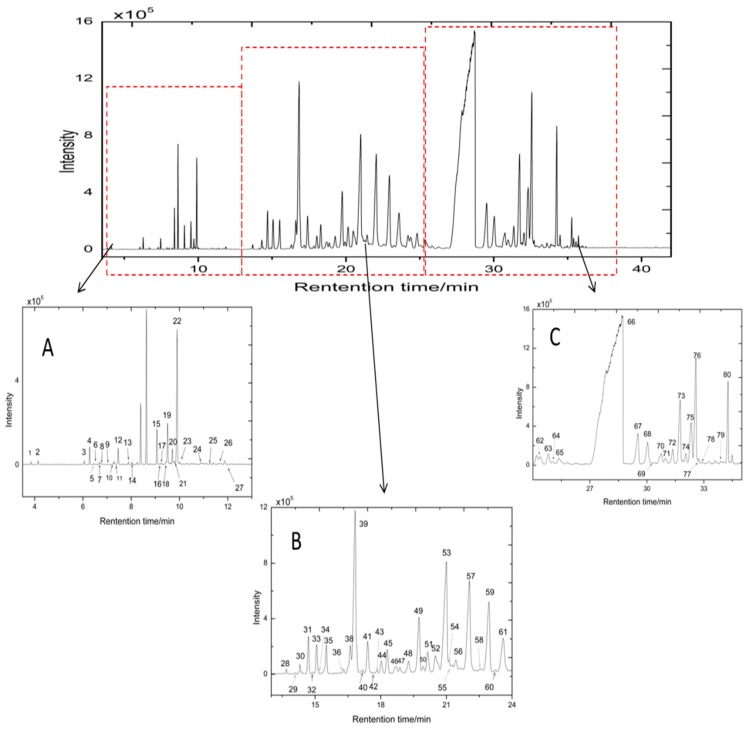
Representative gas chromatography–mass spectrometry (GC-MS) fingerprint of *acorus tatarinowii rhizoma* (ATR): (**A**) total ion chromatogram (TIC) of ATR at 3–13 min; (**B**)TIC of ATR at 13–24 min; (**C**) TIC of ATR at 24–35 min. Eighty volatile components were detected by GC-MS.

The chromatographic fingerprints of 49 ATR samples were obtained by GC-MS analysis and were used to reflect the complexity of volatile chemical components. The representative total ion chromatogram (TIC) of ATR is shown in Figure 1. Eighty components were detected. All GC-MS data, including retention characteristics, peak intensities, and integrated mass spectra, of each sample were used for the qualitative and quantitative analyses. First, the automated mass-spectral deconvolution and identification system (Automatic Mass Spectral Deconvolution and Identification software (AMDIS) software, National Institute of Standards and Technology, Gaithersburg, MD, USA) was used for peak finding and deconvolution. Component identification was based on the comparison of the mass spectrum of putative component with those of the NIST 2005 Mass Spectral Library and NIST 2011. In addition, the chemical components were further identified by temperature-programmed retention indices. The detailed qualitative process was shown in our previous study [15]. Seventy-four volatiles were identified; nine of which, namely, methyleugenol, transmethylisoeugenol, linaool, α-pinene, (–)-terpinen-4-ol, α-terpieol, bornylacetate, α-asarone, and β-asarone, were identified by commercial standard substances. The relative concentration of an individual component was expressed by the ratio of peak area of one component to the internal standard (undecane) on the same TIC. The qualitative and quantitative results are shown in Table S1.

The bioactivities of ATR essential oils were determined by the DPPH radical scavenging assay. The radical scavenging activity was expressed by the equivalent concentration of quercetin. The radical scavenging activities had a maximum value of 41.99 µg/mL, minimum value of 11.60 µg/mL, and mean value of 25.61 µg/mL. The radical scavenging activities of the 49 ATR samples were significantly different. Furthermore, the strongest and the weakest samples are both cultivated in Sichuan province. The result indicated that there is no specific relationship available between the radical scavenging ability and the habitat of the ATR. 

### 2.2. PLSR Model between the Radical Scavenging Activity and the Contents of Volatile Components

**Figure 2:**
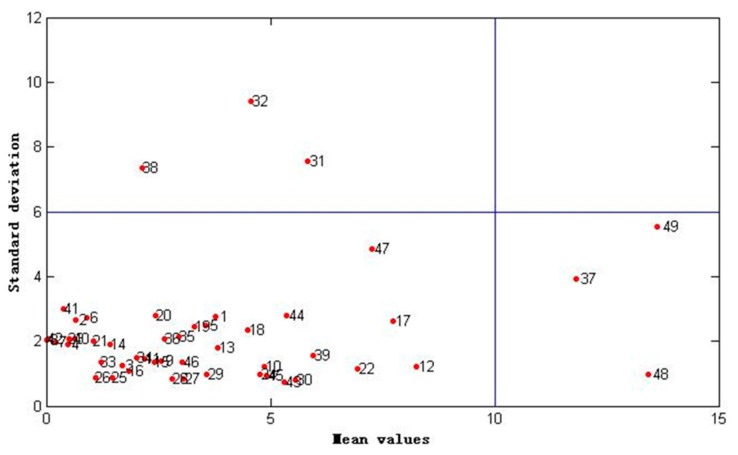
Distribution of the predicted mean and standard deviation values of 2,2-diohenyl-1-picryl-hydrazyl (DPPH) radical scavenging assay for 49 ATR samples by Monte-Carlo cross-validation (MCCV) method. A 2000-time Monte-Carlo sampling was conducted for the dataset of all samples (49 × 80).

Partial least squares regression models were established for the contents of volatile components (matrix X) and the results of DPPH radical scavenging assay (matrix Y) to describe the relationship between the radical scavenging activities and the chemical components. First, the dataset of 49 samples (49 × 80, samples × variables) were used to build the PLS regression model. Thirty-nine samples were selected as the calibration set by using the Kennard–Stone algorithm, and the remaining 10 samples were used as the validation set. For the calibration set, the maximum, minimum, and mean values of the radical scavenging activity were 41.99, 11.60, and 25.97 µg/mL, respectively. For the validation set, the maximum, minimum, and mean values of the radical scavenging activity were 41.27, 14.96, and 24.21 µg/mL, respectively. The distribution of the radical scavenging activity values was similar for the calibration and validation sets. The two datasets were reasonably partitioned. The first eight latent variables were used to establish the PLS regression model as determined by the 10-fold cross-validation. The *R*^2^ and *Q*^2^ values of the model were 0.8284 and 0.7824, respectively, as shown in Table 1. MCCV method was introduced to identify the outliers of the dataset and improve the predictive ability of the regression model. Monte-Carlo sampling was conducted to select 40 ATR data from the original data (49 × 80) to build the PLS regression model. The rest of the data were used as the validation set to evaluate the model. The distribution of the predicted values of DPPH radical scavenging assay for each sample was obtained after sampling 2000 times. The standard deviation and mean predicted values were calculated for each sample as shown in Figure 2. Samples 31, 32, 37, 38, 48, and 49 were identified as outliers in the X-axis and Y-axis directions.

**Figure 3:**
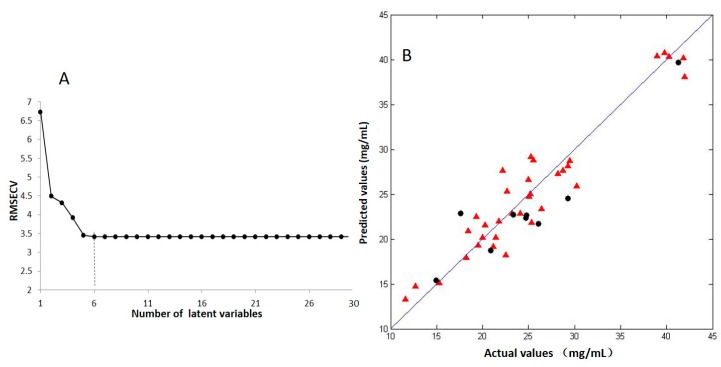
The partial least squares regression model between the radical scavenging activity and the volatile components: (**A**) Selection of the optimal latent variables by 10-fold cross validation. The first six latent variables were selected; (**B**) actual measured DPPH values versus their predicted values obtained by partial least squares regression model. The size of data set is 43 × 80. (▲) calibration set; (●) validation set.

**Figure 4:**
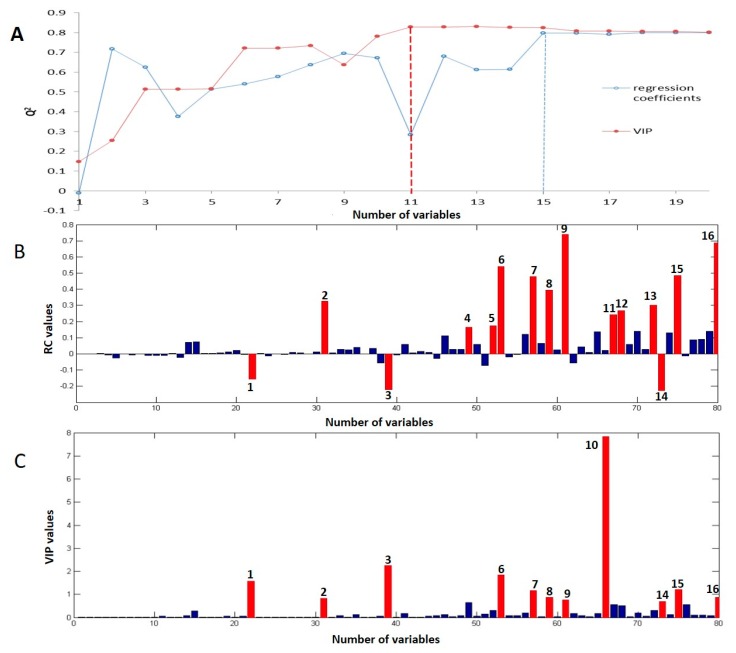
Screening of the key bioactive components: (**A**) The *Q*^2^ of the partial least squares regression models of different combinations of variables. The first one was the variable with the highest variable importance in projection (VIP) or regression coefficient (RC) value. The second combination was the first one plus the second one, then the first three, and so on. In this study, the number of variables changed from one to twenty; (**B**) RCs of the PLS regression model (43 × 80) for the 80 components; (**C**) VIP value of each component. Fifteen and eleven components were selected by RC and VIP, respectively. There are ten common components selected by the two methods. Components **1**–**16**: **1**, Estragole; **2**, Methyleugenol; **3**, cis-Methylisoeugenol; **4**, Shyobunone; **5**, Ledene; **6**, Isoshyobunone; **7**, δ-Cadinene; **8**, Calacorene; **9**, γ-Asarone; **10**, β-Asarone; **11**, *cis*-Calamenene; **12**, Dehydroxy-isocalamendiol; **13**, α-Cadinol; **14**, α-Asarone; **15**, Calamusenone; **16**, Isocalamendiol.

The new dataset (43 × 80) was generated after outlier elimination. Thirty-four samples were selected as the calibration set by the Kennard–Stone algorithm, and the remaining nine samples were used as the validation set. The distribution of radical scavenging activity values was similar before and after the outlier elimination. The new calibration set and validation set were reasonably partitioned. The new PLS regression model is shown in Figure 4B and was established by using the first six latent variables selected by 10-fold cross validation, as shown in Figure 3A. The *R*^2^ and *Q*^2^ values of the model were improved to 0.9090 and 0.8124, respectively, as shown in Table 1. RMSECV decreased from 5.0261 to 3.4109, and RMSEP decreased from 3.2210 to 3.0996. Other parameters for the PLS regression model were compared in Table 1. The results indicated that the efficiency of the model was significantly improved after the outlier elimination. Before the PLS model was selected, both cross validation and external validation was used. The results indicated that the models are not overfitted.

### 2.3. Key Bioactive Components in ATR for Radical Scavenging Activity

**Figure 5:**
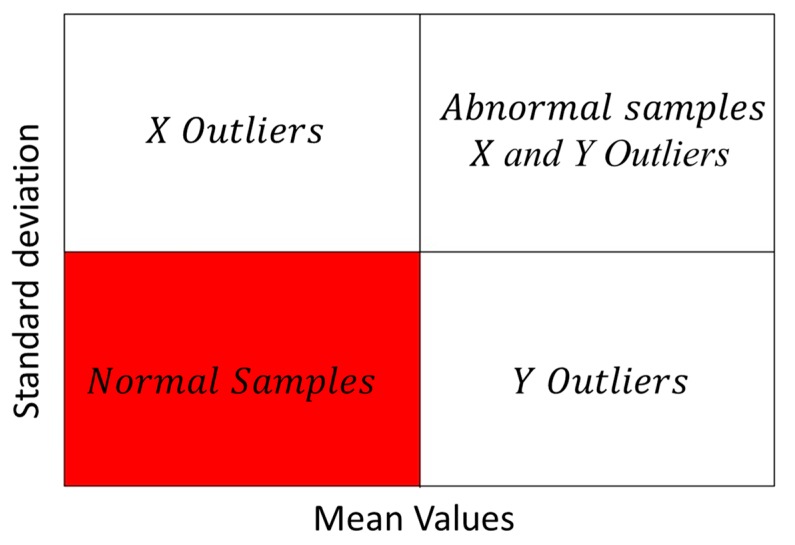
The result of standard deviation versus mean value for each sample calculated by Monte-Carlo cross-validation method. Samples in the red region are identified as normal, whereas the others are outliers.

The present study aims to identify the key components in volatile oils that correspond to the radical scavenging activity of ATR. Thus, the regression coefficient (RC) and variable importance in projection (VIP) methods were employed. A higher absolute value of RC or VIP indicates the bigger contribution of this specific component. These values are scaled and centered, so they are comparable. The RC and VIP values of the 80 components were plotted from the PLSR model (43 × 80) as shown in Figure 4B,C. In addition, combination effect of variables was taken into account in this study [32]. Predictive ability of different variable combinations was compared in order to select the best components’ pattern and help us to define the threshold of variable selection, as shown in Figure 5A. For VIP method, when the number of variables is eleven, *Q*^2^ of the PLSR model obtained the best results, as shown in Figure 5A and Table 1. The results of VIP are better than RC. Thus, the first eleven components were identified as responsible for the radical scavenging activity of ATR volatile oil in this study. They are estragole, methyleugenol, *cis*-methylisoeugenol, isoshyobunone, δ-cadinene, calacorene, γ-asarone, β-asarone, α-asarone, calamusenone, isocalamendiol. Previous studies on ATR volatile oil mainly focused on the two components, α-asarone and β-asarone. These components account for approximately 95% of ATR volatile oils [33]. Thus far, several publications have reported the antioxidant ability of β-asarone, α-asarone [34], and the isoshyobunone, isocalamendiol, and calacorene are the constituents of essential oils with antioxidant activity [35]. The other components are not reported for antioxidant bioactivity, but having other bioactivities. For example, γ-asarone exhibited its fungitoxicity against *Aspergillus flavus* [36]. The isocalamendiol in Zibu Piyin recipe exhibited ameliorating effects on scopolamine-induced memory dysfunction [37]. Calamusenone have insecticidal and repellant activities [38,39]. δ-Cadinene in Psidium cattleianum Sabine has antimicrobial and antioxidant activities [40]. The present study firstly reveals the eleven volatiles as a pattern for predict the radical scavenging activity of ATR. Our findings provide a new focus for the research of bioactivities of ATR.

## 3. Materials and Methods

### 3.1. Materials

Methyleugenol (purity: 98%), trans-methylisoeugenol (purity: 98%), (−)-terpinen-4-ol (purity: 97%), α-terpieol (purity: 97%), bornylacetate (purity: 98%), α-pinene (purity: 98%), undecane (purity: 99%), and linalool (purity: 99%) were obtained from J&K Technology (Beijing, China). β-asarone (purity: 98%), α-asarone (70%), and DPPH were purchased from Sigma-Aldrich (St. Louis, MO, USA). The alkane standard solution of C8–C20 was obtained from Fluka Chemika (Buchs, Switzerland). Quercetin (purity: 99%) was purchased from the National Institute for Food and Drug Control (Beijing, China). Methanol, hexane, and anhydrous sodium sulfate were of analytical grade. Forty-nine batches of ATR samples were deliberately collected from Sichuan, Anhui, Shanxi, Hunan, Hebei, Jiangxi, and Henan Provinces in China. These samples were authenticated by Shao Liu (Xiangya Hospital, Central South University). The specimens are currently preserved in the institute mentioned above.

### 3.2. Extraction of Volatile Oil

All samples were dried at 40 °C for 2 h, followed by pulverization. Volatile oil was extracted according to the procedure described in the Chinese Pharmacopoeia [41]. Briefly, 800 mL of distilled water and 80 g of the sample were added to the standard extractor. The sample was extracted for 4 h with 4 D/min reflux rate. Moderate anhydrous sodium sulfate was added to remove the trace amounts of water. All volatile oils were stored in brown syringes at 4 °C, followed by GC-MS analysis. Undecane was selected as the internal standard in this study.

### 3.3. DPPH Radical Scavenging Assay

The bioactivities of the 49 ATR samples were determined using the DPPH radical scavenging assay. The DPPH radical has a maximum absorbance at 517 nm, which disappeared upon reduction in the presence of antioxidant components in each sample. This phenomenon resulted in a negative correlation between the remaining absorbance at 517 nm and the radical scavenging activity of the sample.

The DPPH assay was conducted as described by Pérez-Meseguer et al. [42] with some modifications. First, the extract was filtered through a 0.45 µm membrane and subsequently diluted 10 times with methanol. The assay mixture comprised ATR extract (0.2 mL) and DPPH solution (41 µg/mL in anhydrous ethanol, 3.8 mL). The quercetin standard solution was used as the positive control, which was diluted with methanol to obtain serial dilutions (5–50 µg/mL). The assay mixture was kept in the dark at room temperature. After 30 min, the absorbance was measured at 517 nm using a UV-Vis spectrophotometer. The radical scavenging activity was calculated by standard curve. DPPH assay and GC-MS analysis were simultaneously conducted to avoid possible variations caused by the degradation of the samples during storage. Each measurement was performed in triplicates. The antioxidant capacity was expressed by the equivalent concentration of quercetin.

### 3.4. GC-MS Analysis

GC-MS analyses were conducted with a Shimadzu GC-2010 gas chromatograph (Kyoto, Japan) coupled with a Shimadzu QP2010 mass spectrometer. The column initial temperature was maintained at 60 °C for 1 min. The column temperature was programmed from 60 to 140 °C at the rate of 5 °C/min, held for 20 min at 140 °C, ramped at a rate of 10 °C/min to 220 °C, and then held for 5 min at 220 °C. The sample (1.0 µL) was injected into a fused-silica capillary column OV-1 (30 m × 0.25 mm with i.d. film thickness of 0.25 µm) with a split ratio of 1:10. The flow rate of the high-purity helium carrier gas was 0.8 mL/min. The injector temperature was 250 °C, and the septum purge flow rate was 3 mL/min. The mass conditions were as follows: interface temperature, 250 °C; ion source temperature, 200 °C; ionization voltage, 70 eV; detector voltage, 0.9 kV; solvent delay, 3 min; and full scan mode in the 35–800 *m*/*z* mass ranges with 0.2/s scan velocities.

Quality control sample was used in this study. For the 49 ATR samples, 10 uL volatile oil of each sample was pooled, then vigorously vortexed for 1 min, to be the quality control sample. The sample injection order is random, one quality control sample after four ATR samples.

### 3.5. Statistical Analysis

#### 3.5.1. Partial Least Squares Regression (PLSR)

PLS can help us to obtain the latent variables, then, build a multivariate linear model between two data matrices, X and Y [43]. This method is very efficient, especially for high dimensional datasets. The PLSR model could be improved by optimizing the number of latent variables. In addition, variable selection is very important to optimize a PLSR model.

In this study, a regression model between the radical scavenging activities and the contents of volatile components was constructed by PLS. Data was centered before the PLS model was developed. Data were partitioned into calibration (80%) and validation (20%) sets using Kennard–Stone algorithm [44]. The number of latent variables were optimized by 10-fold cross validation for the calibration set. The overfitting possibilities of the PLSR model were evaluated by 10-fold cross validation and external validation. The established PLSR models were evaluated using the root mean square error of cross-validation (RMSECV), root mean square error of calibration (RMSEC), root mean square error of prediction (RMSEP), determination coefficient for calibration set (*R*^2^), and determination coefficient for validation set (*Q*^2^) [18,43].

#### 3.5.2. Outlier Identification

Outliers are the data that fall outside the population and were caused by measurement error, sudden changes in experiment conditions, and sample properties [45]. Outliers greatly influence the regression solution, and the existence of such data points might lead to considerable deviations from normality [46]. In this study, the outliers were identified based on Monte-Carlo cross-validation (MCCV) [47]. First, the original data was sampled 2000 times. A fixed percentage (80%) of the samples was randomly selected to build the PLS regression model, and the rest of the data was used to validate the model. The prediction error distribution for each sample was obtained after 2000 times sampling. The distribution of mean value and standard deviation were calculated for each sample. A four-zone scatter plot was established by using the mean value as the coordinate in the X-axis and the standard deviation as the coordinate in the Y-axis, as shown in Figure 5. In this study, 2.5 times of average value (mean value or standard deviation) were selected on the X- and Y-axes to divide the graph into a four-zone scatter plot, according to the suggestion of Reference [38]. PLSR and MCCV were performed using Matlab 7.10.0 software (R2010a, The Math Works Inc., Natick, MA,USA).

## 4. Conclusions

This study proposed an efficient strategy to reveal the key chemical components responsible for the bioactivity of ATR. Chemical components in ATR volatile oils were profiled by GC-MS. A PLSR model between the radical scavenging activity and the contents of volatile components was established. After the outliers were eliminated by MCCV, and informative variables were selected by VIP and RC, the PLSR model was improved significantly and was proven highly reliable. Eleven components, namely, estragole, methyleugenol, cis-methylisoeugenol, isoshyobunone, δ-cadinene, calacorene, γ-asarone, β-asarone, α-asarone, calamusenone, isocalamendiol, were identified using the VIP method. These components formed a pattern and are probably responsible for the radical scavenging activity of ATR and are worthy of further study.

## Figures and Tables

**Table 1:** Comparison of partial least squares regression models before and after variable selection.

Matrix	nLV	*R*^2^	*Q*^2^	RMSEC	RMSEP	RMSECV
49 × 80 ^a^	8	0.8284	0.7824	3.2597	3.2210	5.0261
43 × 80 ^b^	6	0.9090	0.8124	2.3084	3.0996	3.4109
43 × 15 ^c^	5	0.9069	0.7969	2.3440	3.1713	2.9716
43 × 11 ^d^	6	0.8955	0.8284	2.4745	2.9641	3.3196
43 × 10 ^e^	5	0.9050	0.7940	2.3679	3.1932	2.9927

^a^: the PLS regression model established by dataset of all samples; ^b^: the PLS regression model established by dataset of samples after outlier elimination; ^c^: the variables were selected by regression coefficients (RC); ^d^: the variable were selected by variable importance in projection (VIP); ^e^: the common variables selected by RC and VIP. nLV: number of latent variables; *R*^2^: determination coefficient for calibration set; *Q*^2^: determination coefficient for validation set; RMSEC: root mean square error of calibration; RMSEP: root mean square error of prediction (validation set); RMSECV: root mean square error of cross validation.

## Supplementary Materials

Supplementary materials can be found at www.mdpi.com/1422-0067/18/7/1342/s1.

## References

[1] Liao W.P., Li C.P., Yi Y.H., Sun W.W., Gao M.M., Tao S., Yang S.Q. (2005). Study of Antiepileptic Effect of Extracts from Acorus tatarinowii Schott. Epilepsia.

[2] Nandakumar S., Menom S., Shailajan S. (2013). A rapid HPLC-ESI-MS/MS method for determination of β-asarone, a potential anti-epileptic agent, in plasma after oral administration of Acorus calamus extract to rats. Biomed. Chromatogr..

[3] Mukherjee P.K., Kumar V., Mal M., Houghton P.J. (2007). In vitro acetylcholinesterase inhibitory activity of the essential oil from Acorus calamus and its main constituents. Planta Med..

[4] Han P., Han T., Peng W., Wang X.R. (2013). Antidepressant-like effects of essential oil and asarone, a major essential oil component from the rhizome of Acorus tatarinowii. Pharm. Boil..

[5] Wei G., Chen Y.B., Chen D.F., Lai X.P., Liu D.H., Deng R.D., Zhou J.H., Zhang S.H., Li Y.W., Li H. (2013). β-Asarone inhibits neuronal apoptosis via the CaMKII/CREB/Bcl-2 signaling pathway in an in vitro model and AbetaPP/PS1 mice. J. Alzheimer’s Dis..

[6] Yang Y.X., Chen Y.T., Zhou X.J., Hong C.L., Li C.Y., Guo J.Y. (2013). β-asarone, a major component of Acorus tatarinowii Schott, attenuates focal cerebral ischemia induced by middle cerebral artery occlusion in rats. BMC Complement. Altern. Med..

[7] Liu L., Wang L., Shi L., Zhang Z., Du X., Wang Z., Zhang Z. (2013). β-Asarone induces senescence in colorectal cancer cells by inducing lamin B1 expression. Phytomedicine.

[8] Jee Y.L., Jung Y.L., Bongsik Y., Byung K.H. (2004). Antifungal Activity of β-Asarone from Rhizomes of Acorus gramineus. J. Agric. Food Chem..

[9] Zhuang Y.L., Ma Q.Y., Guo Y., Sun L.P. (2017). Protective effects of rambutan (Nephelium lappaceum) peel phenolics on H_2_O_2_-induced oxidative damages in HepG2 cells and d-galactose-induced aging mice. Food Chem. Toxicol..

[10] Loizzo M., Sicari V.I., Tenuta M.C., Leporini M.R., Falco T., Pellicanò T.M., Menichini F., Tundis R. (2016). Phytochemicals content, antioxidant and hypoglycaemic activities of commercial nutmeg mace (*Myristica fragrans* L.) and pimento (*Pimenta dioica*(L.) Merr.). Int. J. Food Sci. Tech..

[11] Huang H.J., Chen H.Y., Chang Y.S., Chen C.C. (2015). Insight into two antioxidants binding to the catalase NADPH binding site from traditional Chinese medicines. RSC Adv..

[12] Wang S.T., Gao W., Fan Y.X., Liu X.G., Liu K., Du Y., Wang L.L., Li H.J., Li P., Yang H. (2016). Phenol profiles and antioxidant capacities of Bistort Rhizoma (*Polygonum bistorta* L.) extracts. RSC Adv..

[13] Liu C., Zhang A.H., Han Y., Lu S.W., Sun H., Yan G.L., Wang P., Wang X.J. (2014). Ultra-High Performance Liquid Chromatography Coupled with Time-of-flight Mass Spectrometry Screening and Analyzing the Potential Bioactive Compounds from Traditional Chinese Medicine Kaixin San, Using Multivariate Data Processing Approach and Metabolynx To. RSC Adv..

[14] Devi S.A., Ganjewala D. (2011). Antioxidant Activities of Methanolic Extracts of Sweet-Flag (Acorus calamus) Leaves and Rhizomes. J. Herbs Spices Med. Plants.

[15] Zhang X.J., Yi L.Z., Deng B.C., Chen L.C., Shi S.T., Zhuang Y.L., Zhang Y. (2015). Discrimination of Acori Tatarinowii Rhizoma and Acori Calami Rhizoma based on quantitative gas chromatographic fingerprints and chemometric methods. J. Sep. Sci..

[16] Yi L.Z., Dong N.P., Liu S., Yi Z.B., Zhang Y. (2014). Chemical features of Pericarpium Citri Reticulatae and Pericarpium Citri Reticulatae Viride revealed by GC-MS metabolomics analysis. Food Chem..

[17] Liu Y., Sun G.X., Luan J.Y., Ling J.H., Zhang J., Yang F.L. (2015). A comprehensive strategy to monitor quality consistency of Weibizhi tablets based on integrated MIR and UV spectroscopic fingerprints, a systematically quantified fingerprint method, antioxidant activities and UPLC-Q-TOF-MS chemical profiling. RSC Adv..

[18] Liu X., Zhan H., Qiao Z., Zheng M., Liu W.Y., Feng F., Yan F.R. (2016). Chemometric analysis based on HPLC multi-wavelength fingerprints for prediction of antioxidant components in Turpiniae Folium. Chemom. Intell. Lab..

[19] Fan X.H., Cheng Y.Y., Ye Z.L., Lin R.C., Qian Z.Z. (2006). Multiple chromatographic fingerprinting and its application to the quality control of herbal medicines. Anal. Chim. Acta.

[20] Xin Z.Q., Ren D.B., Zhang X.J., Yi Z.B., Yi L.Z. (2017). Chromatographic Fingerprints Combined with Chemometric Methods Reveal the Chemical Features of Authentic Radix Polygalae. J. AOAC Int..

[21] Yi L.Z., Yuan D.L., Liang Y.Z., Xie P.S., Zhao Y. (2007). Quality control and discrimination of pericarpium citri reticulatae and pericarpium citri reticulatae viride based on high-performance liquid chromatographic fingerprints and multivariate statistical analysis. Anal. Chim. Acta.

[22] Li L., Zhao Y.Y., Liu W.Y., Feng F., Xie N. (2013). HPLC with quadrupole TOF-MS and chemometrics analysis for the characterization of Folium Turpiniae from different regions. J. Sep. Sci..

[23] Sun Y., Guo T., Sui Y., Li F.M. (2003). Fingerprint analysis of Flos Carthami by capillary electrophoresis. J. Chromatogr. B.

[24] Chaita E., Gikas E., Aligiannis N. (2017). Integrated HPTLC-based Methodology for the Tracing of Bioactive Compounds in Herbal Extracts Employing Multivariate Chemometrics. A Case Study on Morus alba. Phytochem. Anal..

[25] Smilde A., Westerhuis J.A., De J. (2003). A framework for sequential multiblock component methods. J. Chemometr..

[26] Reis M.S. (2013). Network-induced supervised learning: Network-induced classification (NI-C) and network-induced regression (NI-R). AichE J..

[27] Vapnik V. (2000). The Nature of Statistical Learning Theory.

[28] Gad H.A., El-Ahmady S.H., Abou-Shoer M.I., Al-Azizi M.M. (2013). Application of chemometrics in authentication of herbal medicines: A review. Phytochem. Anal..

[29] Kang Q., Ru Q., Liu R., Xu L., Liu J., Wang Y., Zhang Y., Li H., Zhang Q., Wu Q. (2016). On-line monitoring the extract process of Fu-fang Shuanghua oral solution using near infrared spectroscopy and different PLS algorithms. Spectrochim. Acta A.

[30] Lozano V., Peña A., Durán M.I., Mansilla A.E., Escandar G.M. (2013). Four-way multivariate calibration using ultra-fast high-performance liquid chromatography with fluorescence excitation–emission detection. Application to the direct analysis of chlorophylls *a* and *b* and pheophytins *a* and *b* in olive oils. Chemom. Intell. Lab..

[31] Samadi N., Masoum S., Mehrara B., Hosseini H. (2015). Application of linear multivariate calibration techniques to identify the peaks responsible for the antioxidant activity of *Satureja hortensis* L. and *Oliveria decumbens Vent*. essential oils by gas chromatography-mass spectrometry. J. Chromatogr. B.

[32] Yi L.Z., Dong N.P., Shi S.T., Deng B.C., Yun Y.H., Yi Z.B., Zhang Y. (2014). Metabolomic identification of novel biomarkers of nasopharyngeal carcinoma. RSC Adv..

[33] Lam K.Y., Chen J., Lam C.T., Wu Q., Yao P., Dong T.T., Lin H., Tsim K.W. (2016). Asarone from Acori Tatarinowii Rhizoma Potentiates the Nerve Growth Factor-Induced Neuronal Differentiation in Cultured PC12 Cells: A Signaling Mediated by Protein Kinase A. PLoS ONE.

[34] Mukherjee P.K., Kumar V., Mal M., Houghton P.J. (2007). Scientific Validation of Ayurvedic Tradition from Natural Resources. Pharm. Biol..

[35] Lubsandorzhieva P.B., Boldanova N.B., Dashinamzhilov Z.B. (2013). Chemical Composition and In Vitro Antioxidant Activity of Essential Oil from a Hepatoprotective Herbal Mix. Pharm. Chem. J..

[36] Varma J., Tripathi M., Ram V.J., Pandey V.B., Dubey D.K. (2002). γ-Asarone- the fungitoxic principle of the essential oil of Caesulia axillaris. World J. Microbi. Biot..

[37] Zhu L.Y., Lin Z., Zhan L.B., Lu X.G., Peng J.Y., Liang L.N., Yu L., Zheng L.P., Zhang F.L., Liu Q.Q. (2013). The effects of Zibu Piyin Recipe components on scopolamine-induced learning and memory impairment in the mouse. J. Ethnopharmacol..

[38] Chen H.P., Yang K., Zheng L.S., You C.X., Cai Q., Wang C.F. (2015). Repellant and insecticidal activities of shyobunone and isoshyobunone derived from the essential oil of Acorus calamus rhizomes. Pharmacogn. Mag..

[39] Huang Y.Z., Hua H.X., Li S.G., Yang C.J. (2011). Contact and fumigant toxicities of calamusenone isolated from Acorus gramineus rhizome against adults of Sitophilus zeamais and Rhizopertha dominica. Insect Sci..

[40] Scur M.C., Pinto F.G., Pandini J.A., Costa W.F., Leite C.W., Temponi L.G. (2016). Antimicrobial and antioxidant activity of essential oil and different plant extracts of Psidium cattleianum Sabine. Braz. J. Biol..

[41] Chinese Pharmacopoeia Commission (2015). Pharmacopoeia of the People’s Republic of China.

[42] Pérez-Meseguer J., Garza-Juárez A., Salazar-Aranda R., Salazar-Cavazos M.L., de La Torre Rodríguez Y.C., Rivas-Galindo V., Waksman de Torres N. (2010). Development and Validation of an HPLC-DAD Analytical Procedure for Quality Control of Damiana (Turnera diffusa), Using an Antioxidant Marker Isolated from the Plant. J. AOAC Int..

[43] Wold S., Sjöström M., Eriksson L. (2001). PLS-regression: A basic tool of chemometrics. Chemom. Intell. Lab..

[44] Saptoro A., Tadé M.O., Vuthaluru H. (2015). A Modified Kennard-Stone Algorithm for Optimal Division of Data for Developing Artificial Neural Network Models. Chem. Prod. Proc. Model..

[45] Simpson M.B., Bakeev K.A. (2010). Near-Infrared Spectroscopy for Process Analytical Technology: Theory, Technology and Implementation. Process Analytical Technology: Spectroscopic Tools and Implementation Strategies for the Chemical and Pharmaceutical Industries.

[46] Chen D., Shao X., Hu B., Su Q. (2005). Simultaneous wavelength selection and outlier detection in multivariate regression of near-infrared spectra. Anal. Sci..

[47] Cao D.S., Liang L.Z., Xu Q.Z., Li H.D., Chen X. (2009). A new strategy of outlier detection for QSAR/QSPR. J. Comput. Chem..

