# Supplementary material for: GC-MS Fingerprinting Combined with Chemometric Methods Reveals Key Bioactive Components in Acori Tatarinowii Rhizoma
Source: Int J Mol Sci. 2017 Jul 3;18(7):1342. doi: 10.3390/ijms18071342 (PMC5535835; doi:10.3390/ijms18071342)
Supplement: Supplementary file 1 [file ijms-18-01342-s001.zip › 1-ijms-200069-supplementary file/1-ijms-Supplementary materials.docx]

GC-MS Fingerprinting Combined with Chemometric Methods Reveals Key Bioactive Components in Acori Tatarinowii Rhizoma

Wenbin Liu , Bingyang Zhang , Zhongquan Xin , Dabing Ren and Lunzhao Yi *

**Table S1.** The qualitative and quantitative results of essential oils from ATR.

**Listed in ijms-200069-Supplementary materials.**

**Table S2.** Overview of the experimental and predicted DPPH values for PLSR model after outlier elimination

| **Sample ID** | **Y_m_** | **Y_P_** | **RE(%)** |
| --- | --- | --- | --- |
| 1 ^a^ | 11.597 | 13.321 | 14.86 |
| 2 ^a^ | 15.260 | 15.134 | -0.82 |
| 3 ^a^ | 25.038 | 24.777 | -1.04 |
| 4 ^a^ | 28.190 | 27.317 | -3.10 |
| 5 ^a^ | 12.712 | 14.771 | 16.20 |
| 7 ^a^ | 24.102 | 22.859 | -5.16 |
| 8 ^a^ | 25.209 | 25.002 | -0.82 |
| 10 ^a^ | 22.491 | 18.236 | -18.92 |
| 12 ^a^ | 22.183 | 27.653 | 24.66 |
| 13 ^a^ | 24.977 | 26.610 | 6.54 |
| 14 ^a^ | 40.243 | 40.321 | 0.19 |
| 15 ^a^ | 21.406 | 20.171 | -5.77 |
| 16 ^a^ | 21.188 | 19.169 | -9.53 |
| 17 ^a^ | 19.337 | 22.516 | 16.44 |
| 18 ^a^ | 20.269 | 21.570 | 6.42 |
| 19 ^a^ | 29.292 | 28.176 | -3.81 |
| 20 ^a^ | 28.729 | 27.660 | -3.72 |
| 21 ^a^ | 26.409 | 23.337 | -11.64 |
| 22 ^a^ | 22.655 | 25.319 | 11.76 |
| 23 ^a^ | 39.761 | 40.787 | 2.58 |
| 26 ^a^ | 23.224 | 22.842 | -1.64 |
| 29 ^a^ | 25.284 | 21.874 | -13.49 |
| 33 ^a^ | 25.524 | 28.778 | 12.75 |
| 34 ^a^ | 25.279 | 29.189 | 15.46 |
| 36 ^a^ | 41.826 | 40.145 | -4.02 |
| 39 ^a^ | 41.995 | 38.075 | -9.33 |
| 40 ^a^ | 38.966 | 40.410 | 3.71 |
| 41 ^a^ | 19.524 | 19.293 | -1.19 |
| 42 ^a^ | 21.800 | 21.974 | 0.80 |
| 43 ^a^ | 30.243 | 25.931 | -14.26 |
| 44 ^a^ | 29.503 | 28.709 | -2.69 |
| 45 ^a^ | 18.426 | 20.897 | 13.41 |
| 46 ^a^ | 20.013 | 20.151 | 0.69 |
| 47 ^a^ | 18.233 | 17.913 | -1.76 |
| 6 ^b^ | 14.962 | 15.434 | 3.15 |
| 9 ^b^ | 29.259 | 24.554 | -16.08 |
| 11 ^b^ | 20.894 | 18.726 | -10.37 |
| 24 ^b^ | 26.062 | 21.727 | -16.63 |
| 25 ^b^ | 23.370 | 22.706 | -2.84 |
| 27 ^b^ | 24.741 | 22.625 | -8.55 |
| 28 ^b^ | 24.714 | 22.336 | -9.62 |
| 30 ^b^ | 17.636 | 22.887 | 29.77 |
| 35 ^b^ | 41.268 | 39.693 | -3.82 |

^a^: sample in the calibration set; ^b^: sample in the validation set; Y_m_: measured DPPH values; Y_P_: predicted DPPH values; RE: relative error.
